# Bilingualism and Immigration Status Do Not Moderate Family History of Dementia on Cognition among Middle‐Aged Latinx Adults

**DOI:** 10.1002/alz70860_104935

**Published:** 2025-12-23

**Authors:** Lily Kamalyan, Sara E Walker, Alicia R Pacheco, Benjamin D. Huber, Adam Brickman, Jennifer J. Manly

**Affiliations:** ^1^ Columbia University Medical Center, New York, NY, USA; ^2^ Columbia University, New York, NY, USA

## Abstract

**Background:**

Family history of dementia (FHD) increases dementia risk and is associated with greater cognitive impairment in middle‐age. Latinx are at increased risk for Alzheimer's disease and related dementias (ADRDs) compared to non‐Latinx Whites. It is critical to determine dementia risk and protective factors in this vulnerable population. Bilingualism may protect against cognitive aging, but is confounded with language of testing and immigration, the latter of which increases risk for ADRD. We evaluated whether the association of FHD on cognition is moderated by bilingualism, immigration status, and language of testing among middle‐aged Latinx adults.

**Method:**

Participants were 1,121 Latinx community‐dwelling middle‐aged adults (*M*age[SD]=56[10.8], *M*years of education[SD]=12.7[3.6], 72% female, 39% with FHD). Participants completed a comprehensive neuropsychological assessment evaluating the domains of memory, language, processing speed, executive functioning, and visuospatial abilities. Bilingualism was ascertained through a self‐report language history questionnaire. Participants were categorized as monolingual immigrant/Spanish (*n* = 264), bilingual immigrant/Spanish (*n* = 460), bilingual immigrant/English (*n* = 186), and bilingual US born/English (*n* = 211). Multiple linear models evaluated the association between FHD and each cognitive domains across language groups, adjusting for age, education, sex/gender, and parental education.

**Results:**

Spanish‐Monolingual‐Immigrants had the highest rates of FHD (53%), followed by Spanish‐Bilingual‐Immigrants (46%), then English‐Bilingual‐Immigrants (32%) and English‐Bilingual‐US Born (15%). FHD was only associated with worse memory (B=‐0.19,[‐0.35,‐0.03]) and no other domains. Bilingualism was positively associated with processing speed (B=0.15,[0.00,0.31]) while immigration was negatively associated with memory (B=‐0.29,[‐0.53,‐0.06]), language (B=‐0.39,[‐0.56,‐0.22]), and processing speed (B=‐0.31,[‐0.51,‐0.10]). There were no interactions between bilingualism, immigration, or language of testing/bilingual/immigrant group and FHD on any cognitive domain.

**Conclusion:**

Bilingualism and immigration status were independently associated with cognitive functioning. However, neither factor moderated the association of FHD on cognition. Noteworthy, immigration status was negatively associated with more cognitive domains than bilingualism and FHD, suggesting that immigration status might be a more relevant cognitive correlate for our sample of middle‐aged Latinx community members. Evaluating how diverse features of bilingualism (i.e., proficiency, age of acquisition, daily use) may interact with immigration factors (i.e., age of migration, duration of stay, reasons for migration) may clarify their utility in predicting risk for ADRD.

